# Genetic Incorporation of the Favorable Alleles for Three Genes Associated With Spikelet Development in Wheat

**DOI:** 10.3389/fpls.2022.892642

**Published:** 2022-05-03

**Authors:** Xiaojun Zhang, Linyi Qiao, Xin Li, Zujun Yang, Cheng Liu, Huijuan Guo, Jun Zheng, Shuwei Zhang, Lifang Chang, Fang Chen, Juqing Jia, Liuling Yan, Zhijian Chang

**Affiliations:** ^1^State Key Laboratory of Sustainable Dryland Agriculture (in Preparation), College of Agronomy, Shanxi Agricultural University, Taiyuan, China; ^2^Department of Plant and Soil Sciences, Oklahoma State University, Stillwater, OK, United States; ^3^School of Life Sciences and Technology, University of Electronic Science and Technology of China, Chengdu, China; ^4^Crop Research Institute, Shandong Academy of Agricultural Sciences, Jinan, China; ^5^Institute of Wheat Research, Shanxi Agricultural University, Linfen, China

**Keywords:** spikelet node number per spike, spike development, *WFZP* gene, *WAPO* gene, wheat

## Abstract

The number of spikelets per spike is an important trait that directly affects grain yield in wheat. Three quantitative trait loci (QTLs) associated with spikelet nodes per spike (SNS) were mapped in a population of recombinant inbred lines generated from a cross between two advanced breeding lines of winter wheat based on the phenotypic variation evaluated over six locations/years. Two of the three QTLs are *QSns.sxau-2A* at the *WHEATFRIZZY PANICLE* (*WFZP*) loci and *QSns.sxau-7A* at the *WHEAT ORTHOLOG OF APO1* (*WAPO1*) loci. The *WFZP-A1b* allele with a 14-bp deletion at *QSns.sxau-2A* was associated with increased spikelets per spike. *WAPO-A1e*, as a novel allele at *WAPO1*, were regulated at the transcript level that was associated with the SNS trait. The third SNS QTL, *QSns.sxau-7D* on chromosome 7D, was not associated with homoeologous *WAPO-D1* or any other genes known to regulate SNS. The favorable alleles for each of *WZFP-A1*, *WAPO-A1*, and *QSns.sxau-7D* are identified and incorporated to increase up to 3.4 spikelets per spike in the RIL lines. Molecular markers for the alleles were developed. This study has advanced our understanding of the genetic basis of natural variation in spikelet development in wheat.

## Introduction

Wheat (*Triticum aestivum* L., AABBDD, 2*n* = 6*x* = 42) is the most widely grown crop in the world, and its grain is a staple food of humans. Wheat grain yield can be divided into three yield components: spikes per unit of area, grain number per spike, and grain weight (Zhang et al., 2018). Grain number per spike can be subdivided into spikelet number per spike and grain number per spikelet, and the number of spikelets per spike is thus an important subcomponent that can be directly used to improve grain yield (Guo et al., 2015).

Common wheat has a normal spike head that looks a square or “*Q*” (Muramatsu, 1963; Faris et al., 2003). Compared the square spike, a spike that is shorter or compact is called club spike in *T. aestivum* ssp. *Compactum*, whereas a spike that is longer or loose is called spelt spike in *T. aestivum* ssp. *spelta* (Faris et al., 2003; Simons et al., 2006; Johnson et al., 2008; Cui et al., 2012; Faris et al., 2014). The gene controlling the square shape is “*Q*” on chromosomes 5A that was cloned in previous studies (Faris et al., 2003; Simons et al., 2006). The gene controlling the club spike is “*C*,” and the “*C”* is not known yet but was mapped on chromosomes 2D (Johnson et al., 2008; Fan et al., 2019).

A normal wheat spike has one single spikelet on one node of the spike rachis (McMaster, 2005). However, a spike may have a supernumerary spikelet on the same node in a vertical or horizontal pattern, forming twin spikelets and even triple spikelets (Wang et al., 2016). The supernumerary spikelets are generated on the basal and central rachis nodes but not on the upper rachis nodes on the same spike (Sears, 1954). A spike of a normal wheat cultivar may generate 15–25 spikelets, each of which develops as a terminal meristem on the spike (Rawson, 1970). The developed spikelet may advance to differentiate into the floral meristem consisting of 5–10 florets encompassed by two small bract leaves to form fertile spikelets containing grains or may abort to form sterile spikelets without grains (Pennell and Halloran, 1984; Gonzalez et al., 2011; Guo and Schnurbusch, 2015; Sakuma et al., 2019).

Numerous efforts have been made to identify quantitative trait loci (QTLs) for spikelets per spike due to their relatively higher heritability than other grain yield components/subcomponents (Zhang et al., 2018; Kuzay et al., 2019). Many QTLs have been identified in association analyses of wheat panels with various genetic backgrounds (Boeven et al., 2016; Würschum et al., 2018; Voss-Fels et al., 2019; Ward et al., 2019) and biparental populations in both hexaploid and tetraploid wheat (Quarrie et al., 2006; Faris et al., 2014; Xu et al., 2014; Luo et al., 2016; Zhai et al., 2016). In two recent independent studies (Kuzay et al., 2019; Voss-Fels et al., 2019), the *TraesCS7A01G481600* gene on the long arm of chromosome 7A, which is orthologous to *ABERRANT PANICLE ORGANIZATION 1* (*APO1*), known to affect panicle development and spikelet number in rice (Ikeda et al., 2013), was reported to be the candidate gene regulating spikelets per spike in wheat. The *TraesCS7A01G481600* locus was found by fine mapping of segregation populations and association analyses of numerous cultivars.

Supernumerary spikelets are genetically controlled by a single recessive gene (Patil, 1958; Martinek and Bednar, 2001; Aliyeva and Aminov, 2011) or two recessive genes (Sharman, 1967; Millet, 1986; Peng et al., 1998; Yang et al., 2005). A major recessive gene on chromosome 2A and numerous minor genes, including one on chromosome 2B, are reported to be involved in the development of supernumerary spikelets (Klindworth et al., 1990; Zhang et al., 2013). Strong inhibitors of supernumerary spikelets may be located on chromosomes 2DS and 2AL in Chinese Spring (Sears, 1954; Zhang et al., 2013). All of these previous studies have pointed to the homoeologous group 2 chromosomes in the control of supernumerary spikelets in wheat. The wheat *FRIZZY PANICLE* (*WFZP*) gene, which encodes an APETALA2/ethylene response transcription factor, may be involved in supernumerary spikelets in common wheat (Dobrovolskaya et al., 2015). Non-sense mutations in *WFZP-A1* on chromosome arm 2AS and *WFZP-D1* on chromosome arm 2DS cause supernumerary spikelets or a so-called multirow spike (Dobrovolskaya et al., 2017). In addition, the *TB1* gene, which encodes a transcription factor in a class II TCP (TEOSINTE BRANCHED1/CYCLOIDEA/PCF1), may control paired spikelet development in a dosage-dependent manner (Dobrovolskaya et al., 2015; Poursarebani et al., 2015; Dixon et al., 2018).

Previous studies identified the functional genes for spikelet development, laying a foundation for allelic variation in the candidate genes for QTLs for the same traits that are mapped in different populations. In this study, we mapped three major QTLs for spikelet nodes per spike (SNS) in a biparental population in common wheat: the first is associated with *WFZP-A1* on chromosome arm 2AS, the second is associated with *WHEAT ORTHOLOG OF APO1* on chromosome arm 7AL (*WAPO-A1*), and the third is in a previously uncharacterized region on chromosome 7D. These findings have facilitated pyramiding the favorable alleles for the genes/QTLs into a single genotype using molecular markers to increase spikelets per spike in breeding new wheat varieties.

## Materials and Methods

### Plant Materials

“SY95-71” (with a pedigree of Fan6//Fan6/Eronga83) was developed by the Triticeae Research Institute of Sichuan Agricultural University in the 1990s, and “CH7034” (with a pedigree of Jing411/Xiaoyan7430//Zhong8601) was developed by the College of Agronomy of Shanxi Agricultural University. The two advanced breeding lines have obvious differences in spike morphology. A population of 184 F_2:10_ recombinant inbred lines (RILs) derived from the cross SY95-71 × CH7034 was developed to map genes for important traits.

### Phenotypic Identification

The 184 RILs of the SY95-71 × CH7034 population were tested in six locations/years, including Chengdu (104°13′E, 30°47′N) in 2014 and 2015, Yuncheng (110°53′E, 35°00′N) in 2016, and Linfen (111°31′E, 36°54′N) in 2015 and 2016. The field experiments were arranged in a randomized complete block design with three replications in all of the trials. Each line was grown in a single 2-m row with 0.25 m between rows. Twenty seeds were planted in each row with plant spacing of 0.1 m. Nitrogen and superphosphate fertilizers in the six environmental trials were applied at sowing at rates of 120 and 130 kg ha^–1^, respectively. Field management was carried out with standard agronomic practices according to wheat production throughout the growing season to obtain even crop stands (Ma et al., 2019). The number of spikelet nodes per spike was characterized in the main spikes of 15 randomly selected plants from each line at physiological maturity and was calculated as the mean. In addition, three plants from each RIL were planted in the greenhouse of Oklahoma State University in 2019 to determine whether the supernumerary spikelet phenotype was associated with the QTLs for the SNS trait. To avoid any confusion, the number of spikelet nodes per spike in a regular pattern is referred to as SNS, and the total number of spikelets per spike in a pattern with supernumerary spikelets is referred to as SSS.

### Development of Single-Nucleotide Polymorphism Markers

The 184 RILs of the SY95-71/CH7034 population and their parents were genotyped using 17K SNP chips with 17,526 markers and 35K DArT SNP chips containing 36,420 markers at Diversity Arrays Technology Pty Ltd. (Canberra, Australia^1^). The single-nucleotide polymorphism (SNP) marker sequences were used to BLAST the Chinese Spring sequence [International Wheat Genome Consortium (IWGSC) RefSeq v1.1] to determine the physical positions of each marker. The SNP markers that had less than 20% missing values and contained different recombinant information were selected in the final mapping.

### Linkage Group Construction and Quantitative Trait Loci Analysis

The SNP markers were used to construct linkage groups using JoinMap 4.0 (Van-Ooijen, 2006). The genetic distance between markers was estimated using the Kosambi mapping function, and a QTL for SNSs was claimed when the value of the logarithm of the odds (LOD) threshold for significance was above 2.5 in the interval mapping program in MapQTL 6.0 (Van-Ooijen, 2009). A marker with the maximum LOD value was used to determine the chromosomal position of the QTL peak.

### Identification of Allelic Variation in Candidate Genes Associated With Quantitative Trait Loci

Those genes under or close to the peak of the identified QTLs were considered candidates causing the QTLs. Specific primers were designed to isolate and sequence the candidate genes from each of the two parents to determine the allelic variation.

A diagnostic molecular marker for allelic variation in *WFZP-A1* was developed. The primers WFZP-Indel-adF2 (5′-GCCAGCCAACCTCACTTCACTTC-3′) and WFZP-Indel-aR2 (5′-TGGCCGAGGACGCGGCGT-3′) were used to amplify the 449-bp CH7034 allele and the 463-bp SY95-71 allele with the following PCR amplification conditions: 94°C for 3 min, 40 cycles of 94°C for 30 s, 64°C for 30 s, and then 72°C for 40 s, with a final extension of 72°C for 10 min. The PCR products was differentiated on a 2.5% agarose gel at 180 V for 1.5 h.

*WAPO-A1* was isolated using the forward primer WAPO-A1F2 (5′-AGGAGATCAATGTTTTTAGGACAATAGGA-3′) and the reverse primer WAPO-A1R6 (5′-GAGCAGGGTGTAGCAACTAGT-3′) with the following PCR amplification conditions: 94°C for 3 min, 40 cycles of 94°C for 30 s, 58°C for 30 s, and then 72°C for 2 min, with a final extension of 72°C for 10 min. A 2-bp deletion was found to distinguish between the two alleles after the PCR products were digested with restriction enzyme HypCH4V. The primers WAPOA1-Indel-F1 (5′-CACAAACATATTGAATGAAAGTTCC-3′) and WAPOA1-Indel-R3 (5′-CATTATATCTTAAACTATATGATGTCC-3′) were used to amplify a fragment containing the 2-bp deletion with the following PCR amplification conditions: 94°C for 3 min, 38 cycles of 94°C for 30 s, 58°C for 30 s, and then 72°C for 30 s, with a final extension of 72°C for 10 min. The digested PCR products were 42 and 70 bp for the CH7034 allele but 110 bp for the SY95-71 allele. The PCR products were separated and scored on an 8% non-denaturing polyacrylamide gel.

*WAPO-D1* was isolated using the forward primer WAPO-D1F1 (5′-CAGACGCAGAAAGGAAAACCCTAAGGCAACTT-3′) and the reverse primer WAPO-D1R2 (5′-TGCTAAGAACAGAACAACAAATATACTACCC-3′) with the following PCR amplification conditions: 94°C for 3 min, 40 cycles of 94°C for 30 s, 58°C for 30 s, and then 72°C for 3 min, with a final extension of 72°C for 10 min.

### Gene Expression

Quantitative real-time PCR (qRT–PCR) was used to determine the transcriptional levels of *WAPO-*A1 and *WAPO-D1* in SY95-71 and CH7034 cells with SYBR Green PCR Master Mix, and actin was used as an endogenous control. Total RNA was extracted from the leaves using TRIzol^®^ reagent (Invitrogen, Carlsbad, CA, United States). cDNA was synthesized from 1 μg of RNA treated with deoxyribonuclease I using a SuperScript™ II Reverse Transcriptase kit and with an oligo (dT)_20_ primer (Invitrogen). Primers for the gene transcripts were UFO-A-RT-F2 (5′-CTCACTCACTCTCACTCCACG-3′) and UFO-A-RT-R2 (5′-GGTGGTGAGGCAGTAGGTTC-3′) (Kuzay et al., 2019) for *WAPO-A1* and WAPO1-D1-RT-F (5′-CTCACTCTCCCTCCCACCACA-3′) and WAPO1-D1-RT-R (5′-GGTGGTGAGGCAGTAGGTTC-3′) for *WAPO-D1.* qRT–PCR was performed on a CFX96™ Real-time PCR System (Bio–Rad Laboratories, Hercules, CA, United States) using iQTM SYBR^®^ Green Supermix (Bio–Rad Laboratories, Hercules, CA, United States), with actin used as an endogenous control. Gene transcript levels were calculated by the 2^–ΔΔCT^ method, where CT is the threshold cycle.

## Results

### Spikelet Nodes per Spike in Parental Lines and Their Recombinant Inbred Lines

**FIGURE 1:**
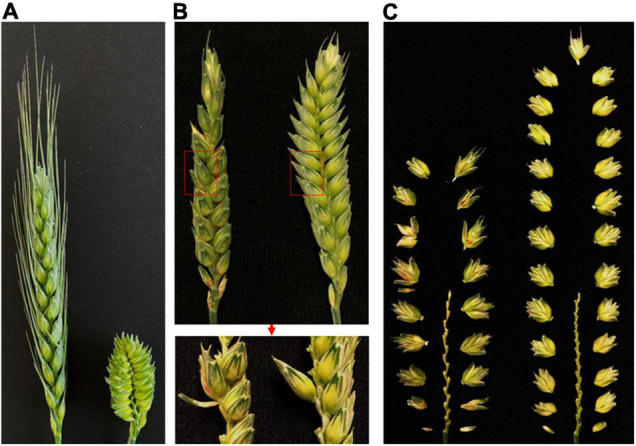
Morphological traits of spikes in SY95-71 and CH7034. **(A)** Comparison of spike between SY95-71 and CH7034. The image shows no supernumerary spikelets in either SY95-71 or CH7034. **(B)** Dissection of supernumerary spikelets. RIL spikes were segregated with supernumerary spikelets (the left side) and without supernumerary spikelets (right). Supernumerary spikelets are shown on spikes within a red square (upper) and dissected spikes (lower). **(C)** Spikelet nodes on spikes. RIL were segregated to have less SNS (left) and more SNS (right). Bars, 1 cm.

No supernumerary spikelet on a spikelet node was observed in either of the two parental lines SY95-71 and CH7034 when tested in different years/locations (Figure 1A). However, supernumerary spikelets were observed in some RILs derived from the two parental lines (Figure 1B), indicating that the supernumerary spikelets were generated due to recombination of genes from the two parental lines. The SNS trait in the SY95-71/CH7034 RIL population showed clear segregation, as illustrated in Figure 1C.

Statistical analysis showed that among the 184 RILs the SNS association under the field conditions was significant (*T* > | *t*_0_._09_|, Supplementary Table 1), indicating that major gene(s) might be responsible for SNS segregation in this population, regardless of the difference in changes in climate or environments in the field.

### Genetic Mapping of Single-Nucleotide Polymorphism Markers in the SY95-71/CH7034 RIL Population

A total of 17,512 SNP markers was eventually generated from the 17K SNP chips, and a total of 36,420 SNP markers was generated from the 35K DArT SNP chips for the 184 RILs of the SY95-71/CH7034 population. According to their sequences, these SNP markers were assembled into 21 chromosomes, forming genetic maps for the RIL population. The total length of the 21 chromosomes containing the 2,347 SNP markers was 4,192 cM, with a marker density of 1.78 cM per marker. Detailed information on the sequence and genetic distances of the SNP markers on the three SNS QTLs mapped in this study is provided in Supplementary Table 2. The SX plus 8-digit SNP codes served as the reference number for each SNP from 17K SNP chips, and the SX-D plus 8-digit SNP codes served as the reference number for each SNP from the 35K DArT chips.

### Allelic Variation in *WFZP-A1* Was Associated With a Spikelet Nodes per Spike Quantitative Trait Locus on Chromosome 2A

**FIGURE 2:**
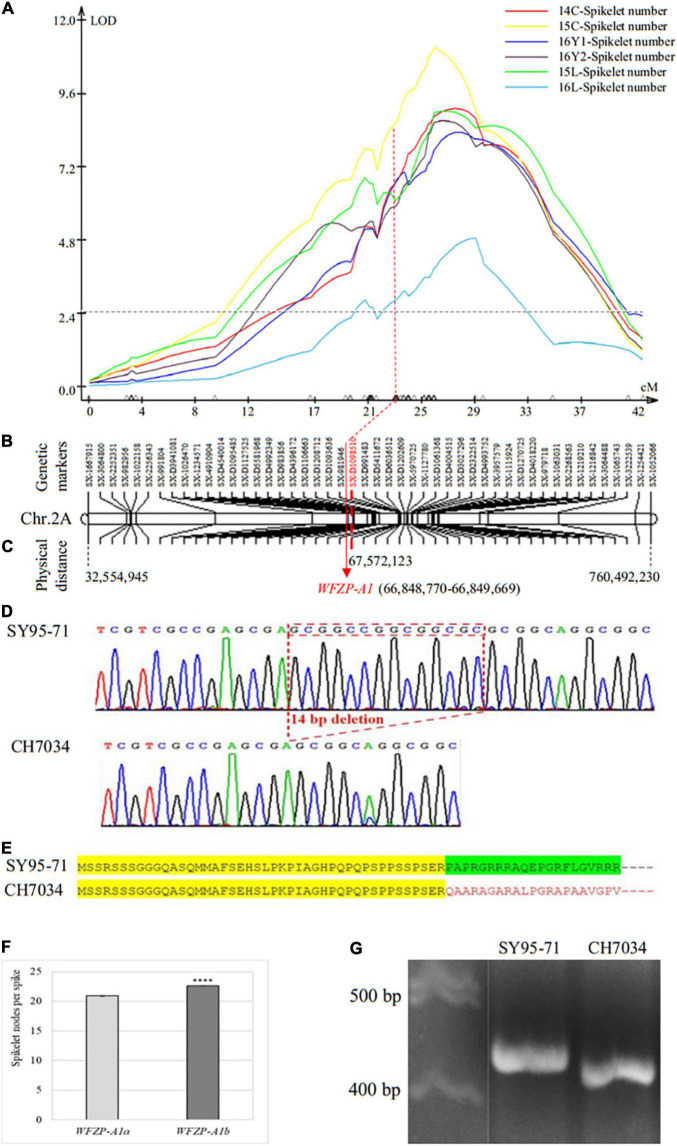
Genetic and physical maps of SNS QTL *QSns.sxau-2A* and allelic variation in the candidate gene *WFZP-A1*. **(A)** Mapping of the *QSns.sxau-2A* locus. The phenotypic data was collected in Chengdu in 2014 (14C) and 2015 (15C), two experimental locations in Yuncheng in 2016 (16Y1 and 16Y2), and Linfen in 2015 (15L) and 2016 (16L). LOD value is indicated on Y axis, and genetic distance (cM) is indicated on X axis. **(B)** Genetic map of *QSns.sxau-2A*. The SX plus 8-digit SNP codes are markers from SNP from 17K SNP chips, and the SX-D plus 8-digit SNP codes are markers from the 35K DArT chips. **(C)** Physical map of *QSns.sxau-2A*. The physical locations of markers and candidate gene *WFZP-A1* are shown based on the Chinese Spring genome sequence of RefSeq v1.1. **(D)** Allelic variation in *WFZP-A1.* The 14-bp sequence that is placed in a red square is present in SY95-71 but absent in CH7034. **(E)** Deduced WFZP-A1 proteins in SY95-71 and CH7034. Conserved amino acids before the deletion in both SY95-71 and CH7034 are highlighted in yellow, functional amino acids after the deletion in SY95-71 are highlighted in green, and altered amino acids after the deletion in CH7034 are indicated in red. Dots indicate more amino acids toward to C terminus. **(F)** Comparison of SNS between the SY95-71 allele and the CH7034 allele. A *t*-test was performed to show significant level in SNS between the two alleles. The bars indicate standard error. “****” indicates significant difference (*p* < 0.0001). **(G)** A diagnostic PCR marker for *WFZP-A1* between the SY95-71 and CH7034. The marker was developed to distinguish between the SY95-71 allele without the 14-bp deletion and the CH7034 allele with the 14-bp deletion.

A total of 1,738 SNP markers, including 797 from 17K SNP chips and 941 from 35K DArT SNP chips, were assembled into chromosome 2A, which was confirmed by the IWGSC RefSeq v1.1 sequences. On the basis of whole-chromosome QTL scanning using interval mapping (IM) analysis, a QTL, *QSns.sxau-2A*, was found on this chromosome (Figure 2A). The LOD value at the peak position of *QSns.sxau-2A* ranged from 4.89 to 11.13 in six experimental locations/years, and this QTL alone explained 11.65 or 24.20% of the total phenotypic variation. The 48 markers on chromosome 2A were selected as a linkage group to indicate the genetic distance and physical location of the *QSns.sxau-2A* locus (Figure 2B).

The *WFZP-A1* gene at position 66,848 kb was close to marker *SX-D1098510*, which was under the peak of *QSns.sxau-2A* (Figure 2C); hence, *WFZP-A1* was considered a candidate gene causing *QSns.sxau-2A*. Sequencing results revealed that the SY95-71 allele showed the same sequence as the *WFZP-A1* gene of Chinese Spring, which is referred to as the *WFZP-A1a* allele, whereas the CH7034 allele had a 14-bp deletion in its coding region, which is referred to as the *WFZP-A1b* allele (Figure 2D). The 14-bp insertion in the *WFZP-A1a* allele could cause it to gain its function at the protein level, or the 14-bp deletion in the *WFZP-A1b* allele could cause it to lose its function at the protein level (Figure 2E). No association was found between the *WFZP-A1* marker and the SSS trait in this study.

On average, over the six experimental locations/years, the CH7034 *WFZP-A1b* allele (22.6 ± 0.1, *n* = 593) set 1.7 more spikelet nodes per spike than the SY95-71 *WFZP-A1a* allele (20.9 ± 0.1, *n* = 450) at the *QSns.sxau-2A* locus (*p* = 8.68E-27) (Figure 2F). Based on the 14-bp indel, a diagnostic marker for *WFZP-A1* was developed (Figure 2G).

### Allelic Variation in *WAPO-A1* Was Associated With a Spikelet Nodes per Spike Quantitative Trait Locus on Chromosome 7A

**FIGURE 3:**
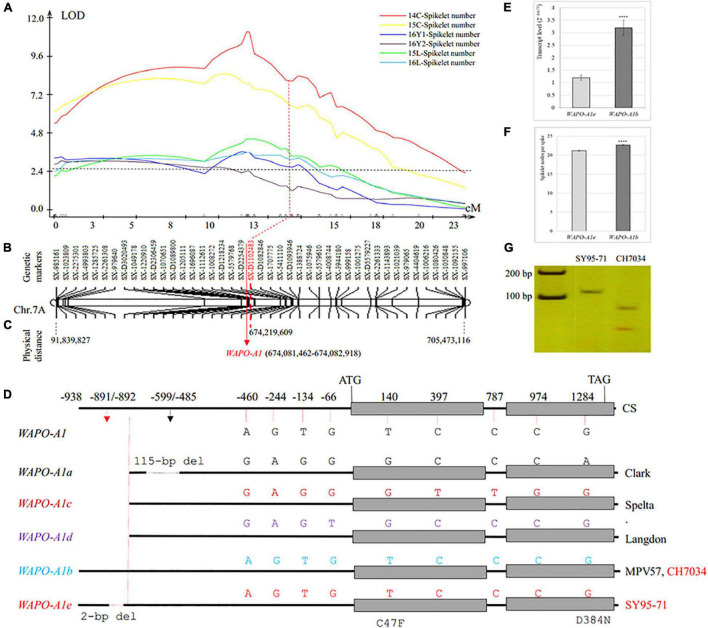
Genetic and physical maps of SNS QTL *QSns.sxau-7A* and allelic variation in the candidate gene *WAPO-A1*. **(A)** Mapping of the *QSns.sxau-7A* locus. The phenotypic data was collected in Chengdu in 2014 (14C) and 2015 (15C), two experimental locations in Yuncheng in 2016 (16Y1 and 16Y2), and Linfen in 2015 (15L) and 2016 (16L). LOD value is indicated on Y axis, and genetic distance (cM) is indicated on X axis. **(B)** Genetic map of *QSns.sxau-7A*. The SX plus 8-digit SNP codes are markers from SNP from 17K SNP chips, and the SX-D plus 8-digit SNP codes are markers from the 35K DArT chips. **(C)** Physical map of *QSns.sxau-7A*. The physical locations of markers and candidate gene *WAPO-A1* are shown based on the Chinese Spring genome sequence of RefSeq v1.1. **(D)** A diagram of differences in multiple *WAPO-A1* alleles. Allelic variation in *WAPO-A1a* through *WAPO-A1d* is cited from the previous study (Kuzay et al., 2019). CH7034 has the same *WAPO-A1b* allele as MPV57. SY95-71 has *WAPO-A1e*, which is a novel allele. The only difference between *WAPO-A1b* and *WAPO-A1e* is that *WAPO-A1e* has a 2-bp deletion in its promoter. **(E)** Comparison of transcript levels between the SY95-71 *WAPO-A1e* allele and the CH7034 *WAPO-A1b* allele. **(F)** Comparison of SNS between the SY95-71 *WAPO-A1e* allele and the CH7034 *WAPO-A1b* allele. **(E–F)** A *t*-test was performed to show differences in the transcript levels between the two alleles, with “****” indicating a significant difference (*p* < 0.0001). The bars indicate standard error. **(G)** A diagnostic PCR marker for *WAPO-A1* between the SY95-71 and CH7034. The PCR products were digested with restriction enzyme HypCH4V to distinguish between the SY95-71 *WAPO-A1e* allele with the 2-bp deletion and the CH7034 *WAPO-A1b* allele without the 2-bp deletion.

A QTL analysis revealed that SNS was associated with 65 markers on chromosome 7A (*QSns.sxau-7A*). The 1,730 SNP markers including 844 from 17K SNP chips and 886 from 35K DArT SNP chips were assembled into chromosome 7A, but only 43 markers on chromosome arm 7AL were selected as a linkage group to indicate the genetic distance and physical location of the *QSns.sxau-7A* locus (Figure 3A). The LOD values at the peak position of *QSns.sxau-7A* ranged from 3.20 to 11.00 in five experimental locations/years, and this QTL alone explained 9.17–28.18% of the total phenotypic variation.

The *WAPO-A1* gene (*TraesCS7A02G481600*) was considered a candidate gene causing *QSns.sxau-7A*, as it was close to marker *SX-D1102483* that was under the peak of *QSns.sxau-7A* (Figures 3B,C). Primers that were reported to specifically amplify *WAPO-A1* (Kuzay et al., 2019) were used to isolate this gene from two parental lines. No difference was found between the CH7034 and *WAPO-A1b* alleles in the sequenced 2,439-bp region, including the 938-bp promoter region, 1,457 bp from the start codon to the stop codon, and 42 bp at the 3′ end of the gene. Compared with CH7034, SY95-71 had a 2-bp deletion at positions –891 to –892 bp, which was only the difference between the two parental lines (Figure 3D). Compared with the four reported alleles (*WAPO-A1a* through *-A1d*), the *WAPO-A1* allele in SY95-71 was novel and was thus named *WAPO-A1e* (Figure 3D).

The *WAPO-A1* transcript levels in 1-cm young spikes were compared between the *WAPO-A1b* and *WAPO-A1e* alleles. The CH7034 *WAPO-A1b* allele showed 2.6 times higher transcript levels than the *WAPO-A1e* allele (*p* = 0.0001) (Figure 3E). This result suggests that either the *WAPOA1b* promoter might increase its transcript levels due to the absence of the 2-bp deletion or the *WAPO-A1e* promoter reduces its transcript levels due to the presence of the 2-bp deletion.

The CH7034 *WAPO-A1b* allele (22.7 ± 0.1, *n* = 554) showed 1.5 more spikelet nodes per spike than the SY95-71 *WAPO-A1e* allele (21.2 ± 0.1, *n* = 435) at the *QSns.sxau-7A* locus (*p* = 1.54E-19), based on the averaged SNS from the six experimental locations/years (Figure 3F). The results showed an association of the SNS phenotype with the 2-bp deletion genotype. A diagnostic marker was developed to distinguish *WAPO-A1e* that had the 2-bp deletion from *WAPO-A1b* that did not have the 2-bp deletion (Figure 3G).

### A New Spikelet Nodes per Spike Quantitative Trait Locus on Chromosome Arm 7DL

**FIGURE 4:**
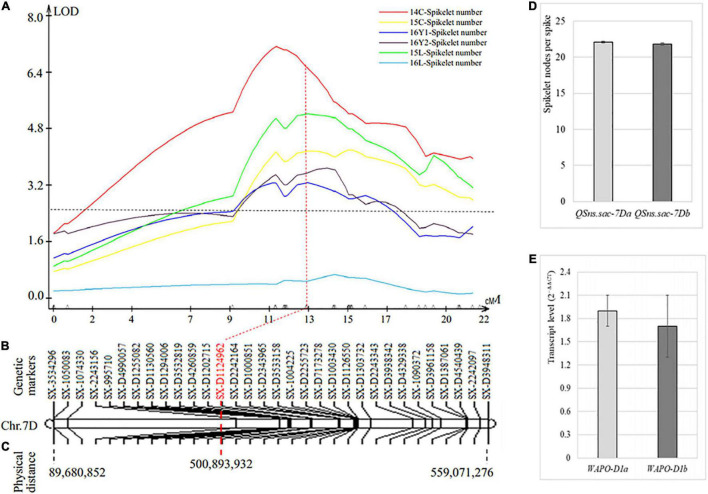
Genetic and physical maps of SNS QTL *QSns.sxau-7D*. **(A)** Mapping of the *QSns.sxau-7D* locus. The phenotypic data was collected in Chengdu in 2014 (14C) and 2015 (15C), two experimental locations in Yuncheng in 2016 (16Y1 and 16Y2), and Linfen in 2015 (15L) and 2016 (16L). LOD value is indicated on Y axis, and genetic distance (cM) is indicated on X axis. **(B)** Genetic map of *QSns.sxau-7D*. The SX plus 8-digit SNP codes are markers from SNP from 17K SNP chips, and the SX-D plus 8-digit SNP codes are markers from the 35K DArT chips. **(C)** Physical map of *QSns.sxau-7D*. The physical locations of markers are shown based on the Chinese Spring genome sequence of RefSeq v1.1. **(D)** Comparison of SNS between the SY95-71 *QSns.sxau-7D* allele and the CH7034 *QSns.sxau-7D* allele. **(E)** Comparison of *WAPO-D1* transcript levels between the SY95-71 and the CH7034, without significant difference.

The third SNS QTL was located on chromosome 7D (*QSns.sxau-7D*), where only SNP markers, including 8 from 17K SNP chips and 24 from 35K DArT SNP chips, were assembled into the whole chromosome, spanning 22 cM in genetic distance (Figure 4A). *QSns.sxau-7D* explained up to 17.92% of the total phenotypic variation in five of the six experimental locations/years, and the effect was not significant in one experimental location/year. The marker *SX-D1124962* closest to the peak of *QSns.sxau-7D* was associated with the SNS trait, the SY95-71 allele (22.1 ± 0.1, *n* = 561) versus the CH7034 allele (21.8 ± 0.1, *n* = 536) (*p* = 0.091) (Figures 4B–D).

The complete gene of homoeologous *WAPO-D1* on chromosome 7D (*TraesCS7D02G468700*) was isolated and sequenced, and the sequenced region, including 564 bp upstream from the start codon, 1,488 bp from the start codon to the stop codon, and 196 bp at the 3′ end of the gene, showed no difference between the SY95-71 and CH7034 alleles. Both SY95-71 and CH7034 had the same *WAPO-D1* sequence as Chinese Spring. *WAPO-D1* showed no significant difference in its transcript level between the two alleles (Figure 4E). The SNP at *SX-D1124962* may be the closest marker to the gene causing *QSns.sxau-7D.*

### Allele Combinations of the Three Spikelet Nodes per Spike Quantitative Trait Loci

Further analyses were performed on each of the three SNS QTLs as independent genetic factors. Eight genotypes generated from these three genes/QTLs, each with two alleles, were analyzed for their SNS phenotypes.

**FIGURE 5:**
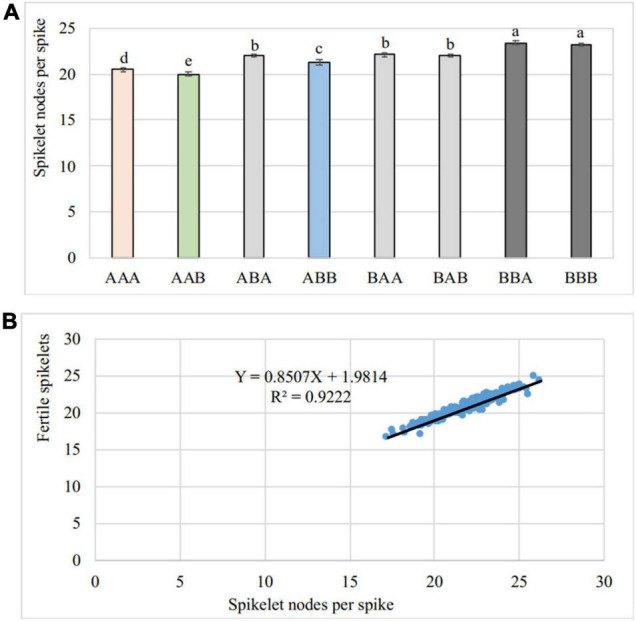
Overall analyses of SNS and fertile spikelets. **(A)** Comparison of SNS among eight genotypes. These eight genotypes are in the order of *WFZP-A1*, *WAPO-A1*, and *QSns.sxau-7D*, with A for the SY95-71 allele and B for the CH7034 allele. The averaged SNS over the six experimental locations were comparisons. A *t*-test was performed to show significant levels in difference of SNS between the two alleles, with *a* though *e* for a significant difference (*P* < 0.05). **(B)** Correlation of SNS and fertile spikelets. The averaged SNS and fertile spikelets over the six experimental locations were analyzed their correlations.

These eight genotypes were classified for the three QTLs in the order of *WFZP-A1*, *WAPO-A1*, and *QSns.sxau-7D*. The SY95-71 allele was designated A, and the CH7034 allele was designated B. The contribution of each genotype to the phenotype was assessed by comparing the SNS mean, given the lack of epistatic interactions. The genotype expected to generate the highest SNS should be the recombinant (B_B_A), containing all the *WFZP-A1b* and *WAPO-A1b* alleles from CH7034 and *QSns.sxau-7Da* from SY95-71, whereas the genotype expected to generate the lowest SNS should be the recombinant (A_A_B), containing all the *WFZP-A1a* and *WAPO-A1e* alleles from SY95-71 and *QSns.sxau-7Da* from CH7034. The incorporated B_B_A genotype generated 23.4 ± 0.2 (*n* = 149), which was 3.4 more spikelets than the incorporated A_A_B genotype 20 ± 0.2 (*n* = 124). The remaining six genotypes showed intermediate SNS scores (Figure 5A).

The recently released genome sequences of 10 wheat cultivars provided haplotype information for the frequencies of favorable alleles, *WFZP-A1* and *WAPO-A1*, from CH7034. Only cultivar Norin61 had the same *WFZP-A1b* allele with the 14-bp deletion as CH7034. All other cultivars showed 100% identity to SY95-71 without the 14-bp deletion, except Spelt, which had a SNP in the *WFZP-A* gene region.

CH7034 showed 100% identity in the complete 2,439-bp sequence of the *WAPO-A1b* allele as three wheat cultivars: Mace, Norin61, and Stanley. None of these ten cultivars showed 100% identity to the complete 2,437 bp sequence of the SY95-71 *WAPO1-A1e* allele, but the 2-bp deletion in *WAPO1-A1e* was found to exist in six wheat cultivars: Arina, Jaggar, Julius, Lancer, Landmark, and SY_Mattis. These cultivars were found to have the *WAPO1-A1a* allele, but the sequence of the *WAPO1-A1a* allele did not include the 2-bp deletion region identified in a previous study (Figure 3D; Kuzay et al., 2019; Voss-Fels et al., 2019). The last cultivar, Spelt, had no 2-bp deletion, but had a *WAPO1-A1* that was different from *WAPO1-A1a* through *WAPO1-A1e.*

### Relationships Between the Total Spikelets, Fertile Spikelets, and Sterile Spikelets

Correlation analysis examined the correlation of the total SNS with fertile spikelets per spike and sterile spikelets per spike over the six experimental locations/years. The total SNS was positively correlated with fertile spikelets per spike (*R*^2^ = 0.922, *n* = 184) (Figure 5B). The results indicated that fertile spikelets were increased through spikelet development in wheat.

## Discussion

Allelic variations in the two advancing lines were identified being associated with three QTLs for SNS. Among the three SNS QTLs identified in this study, *QSns.sxau-2A* was most stably and consistently observed under six different experimental locations/years, but *QSns.sxau-7D* was mostly regulated by the environments. The study provides genetic information for grains per spike that can be increased through regular spikelets per spike, in addition to the spikelet fertility rate and grain number per spikelet in wheat (Guo et al., 2015; Zhang et al., 2018).

Previous studies have suggested that mutants in *WFZP* genes are important resources for increasing supernumerary spikelets to increase grains per spike in bread wheat (Dobrovolskaya et al., 2016; Dobrovolskaya et al., 2017; Zhang et al., 2017; Du et al., 2021; Li et al., 2021). While *WFZP-D1* lesions were combined with a frame shift mutation in *WFZP-A1* due to the 14-bp deletion, supernumerary spikelets were detectable. In this study, the CH7034 *WFZP-A1b* allele was not associated with the supernumerary spikelet trait that was observed in the segregation population. No QTL for supernumerary spikelets was observed in the orthologous *WFZP-D* region or any other genomic region. These results indicated that only the CH7034 *WFZP-A1b* allele was unable to generate supernumerary spikelets and that the supernumerary spikelet trait may be regulated by other unknown genetic factors in common wheat. Surprisingly, the CH7034 *WFZP-A1b* allele with the 14-bp deletion was found to positively regulate the SNS number. This observation suggested that wild-type *WFZP-A1a* might be a repressor of spikelet development, although the possibility that mutated *WFZP-A1b* may gain new function due to the presence of deduced new amino acids cannot be excluded. Gene editing technology can be used to edit three homoeologous *WFZP* genes to increase grain number to improve bread wheat yield in future studies. *WAPO1* on wheat chromosome arm 7AL was identified as the best candidate gene regulating spikelet number per spike based on a fine genetic map, a genome-wide association analysis, and the known functions of the orthologous *APO1* gene in rice (Kuzay et al., 2019; Voss-Fels et al., 2019). Since then, more robust evidence from numerous materials with various genetic backgrounds that were tested under different environments and from mutants and transgenic wheat (Ma et al., 2019; Ding et al., 2021; Jones et al., 2021; Kuzay et al., 2022) has demonstrated that *WAPO-A1* is the causal gene for the previously reported QTLs. *WAPO-A1b* in haplotypes such as Berkut had more than threefold higher transcript levels than *WAPO-A1a* with the 115-bp deletion and three SNPs in the promoter in haplotypes such as RAC875, suggesting that the reduced transcript level of *WAPO-A1a* was due to the 115-bp deletion and three SNPs (Kuzay et al., 2019; Voss-Fels et al., 2019). A previous study also suggested that the reduced SNS by *WAPO-A1a* may be due to the effects of reduced transcript levels or two point mutations in its protein (C47F and D384N) that cannot be separated in the experiment. In this study, *WAPO-A1b* and *WAPO-A1e* encoded the same protein sequence but showed differences in their effects on the SNS trait, excluding the possibility that the SNS trait was regulated at the protein level.

*WAPO-A1b* and *WAPO-A1e* showed differences at the transcript level, indicating that the SNS trait was regulated at the transcript level. *WAPO-A1e* in SY95-71 has the same DNA sequence as *WAPO-A1b* that does not have the previously reported 115-bp deletion or three SNP regions in CH7034. However, further sequencing showed that *WAPO-A1e* had a 2-bp deletion in upstream of the promoter, facilitating mapping of *WAPO-A1* in this study. It is noteworthy that *WAPO-A1* in another SY95-71 showed the 115-bp deletion (Ma et al., 2019). It is likely that the two SY95-71 breeding lines might have the same source but different genetic backgrounds that were segregated in progeny plants. *WAPO-A1b* in CH7034 showed higher transcript levels than *WAPO-A1e* with the 2-bp deletion in the promoter in SY95-71, suggesting that the transcript level of *WAPO-A1e* was reduced, as observed in *WAPO-A1a*. It is likely that the promoter is involved not only in the previously reported 115-bp deletion and three SNP regions ranging from positions –599 to –485 but also in the 2-bp deletion at positions –891 and –892. Further sequencing results at positions up to –2,565 bp revealed seven SNPs further upstream, including six SNPs at positions 2,210, 2,217, 2,243, 2,255, 2,272, and 2,341 bp between the SY95-71 and CH7034 alleles at *WAPO-A1.* Further work is needed to identify actual binding sites in the *WAPO1* promoter.

The tight association between the two SNS QTLs and two genes, *WFZP-A1* and *WAPO-A1* involved in spikelet development, suggests that each of the spikelet development genes was considered as a candidate gene for one SNS QTL. However, the candidate genes need to be validated in large populations. In the present study the limited size of the RIL population was used for mapping only. Specific RILs can be selected to develop backcross populations, in which only one locus of the three QTLs is heterozygous while the other two are fixed for the same allele; therefore, each of these backcross populations can be used to clone each of the three QTLs in future projects. Diagnostic markers for the *WFZP-A1b* allele, which should be selected, and the *WAPO1e* allele, which should be selected against, will provide molecular tools to support the modification of wheat spikes in wheat breeding.

## Conclusion

In summary, this study identified allelic variation in three QTLs associated with SNS in the same mapping population, two of which were associated with the genes known to involve in spikelet development in wheat. The findings that *WFZP-A1b* with the 14-bp deletion increased spikelets per spike and the *WFZP-A1e* as a novel allele was regulated at the transcript level advanced understanding of these known genes. The molecular markers for these allelic variations can be used to incorporate and pyramid the favorable alleles into single lines for breeding of new varieties. Improved understanding of the genetic basis of natural variation in the spikelet development will be particularly important to wheat production worldwide.

## Data Availability Statement

The original contributions presented in the study are included in the article/Supplementary Material, further inquiries can be directed to the corresponding authors.

## Author Contributions

XZ and LQ performed most of the phenotyping and molecular experiments. XL, CL, HG, SZ, LC, FC, and JJ undertook part of the field and laboratory work. ZY participated in the construction of the RILs. JZ contributed the RILs’ phenotypic data in Linfen. ZC designed and directed the field experiments. LY directed molecular research work. LY, ZC, and LQ wrote the manuscript. All authors contributed to the article and approved the submitted version.

## Conflict of Interest

The authors declare that the research was conducted in the absence of any commercial or financial relationships that could be construed as a potential conflict of interest. The handling editor declared a past co-authorship with one of the authors CL.

## Publisher’s Note

All claims expressed in this article are solely those of the authors and do not necessarily represent those of their affiliated organizations, or those of the publisher, the editors and the reviewers. Any product that may be evaluated in this article, or claim that may be made by its manufacturer, is not guaranteed or endorsed by the publisher.

## Footnotes

1
https://www.diversityarrays.com

## References

[B1] Aliyeva A. J. Aminov N. K. (2011). Inheritance of the branching in hybrid populations among tetraploid wheat species and the new branched spike line 166-schakheli. *Genet. Resour. Crop Evol.* 58 621–628. 10.1007/s10722-011-9702-9

[B2] Boeven P. H. G. Longin C. F. H. Leiser W. L. Kollers S. Ebmeyer E. Würschum T. (2016). Genetic architecture of male floral traits required for hybrid wheat breeding. *Theor. Appl. Genet.* 129 2343–2357. 10.1007/s00122-016-2771-6 27553082

[B3] Cui F. Ding A. Li J. Zhao C. Wang L. Wang X. (2012). QTL detection of seven spike-related traits and their genetic correlations in wheat using two related RIL populations. *Euphytica* 186 177–192. 10.1007/s10681-011-0550-7

[B4] Ding P. Y. Zhou J. G. Zhao C. H. Tang H. P. Mu Y. Tang L. W. (2021). Dissection of haplotypes, geographical distribution and breeding utilization of WAPO1 associated with spike development in wheat. *Acta. Agron. Sin.* 11 1–16.

[B5] Dixon L. E. Greenwood J. R. Bencivenga S. Zhang P. Cockram J. Mellers G. (2018). TEOSINTE BRANCHED1 regulates inforescence architecture and development in bread wheat (*Triticum aestivum*). *Plant Cell* 30 563–581. 10.1105/tpc.17.00961 29444813 PMC5894836

[B6] Dobrovolskaya O. B. Amagai Y. Popova K. I. Dresvyannikova A. E. Martinek P. Krasnikov A. A. (2017). Genes wheat frizzy panicle and sham ramification 2 independently regulate differentiation of floral meristems in wheat. *BMC Plant Biol.* 17:252. 10.1186/s12870-017-1191-3 29297328 PMC5751757

[B7] Dobrovolskaya O. B. Pont C. Orlov Y. L. Salse J. (2016). Development of new SSR markers for homoeologous WFZP gene loci based on the study of the structure and location of microsatellites in gene rich regions of chromosomes 2AS, 2BS, and 2DS in bread wheat. *Russ. J. Genet.* 19 330–337. 10.1134/S2079059716030023

[B8] Dobrovolskaya O. B. Pont C. Sibout R. Martinek P. Badaeva E. Murat F. (2015). FRIZZY PANICLE drives supernumerary spikelets in bread wheat. *Plant Physiol.* 167 189–199. 10.1104/pp.114.250043 25398545 PMC4281007

[B9] Du D. J. Zhang D. X. Yuan J. Feng M. Li Z. J. Wang Z. H. (2021). FRIZZY PANICLE defines a regulatory hub for simultaneously controlling spikelet formation and awn elongation in bread wheat. *New Phytol.* 231 814–833. 10.1111/nph.17388 33837555

[B10] Fan X. L. Cui F. Ji J. Zhang W. Zhao X. Q. Liu J. J. (2019). Dissection of pleiotropic QTL regions controlling wheat spike characteristics under different nitrogen treatments using traditional and conditional QTL mapping. *Front. Plant Sci.* 10:187. 10.3389/fpls.2019.00187 30863417 PMC6400075

[B11] Faris J. D. Fellers J. P. Brooks S. A. Gill B. S. (2003). A bacterial artificial chromosome contig spanning the major domestication locus Q in wheat and identification of a candidate gene. *Genetics* 164 311–321. 10.1093/genetics/164.1.311 12750342 PMC1462558

[B12] Faris J. D. Zhang Q. J. Chao S. M. Zhang Z. C. Xu S. S. (2014). Analysis of agronomic and domestication traits in a durum × cultivated emmer wheat population using a high-density single nucleotide polymorphism-based linkage map. *Theor. Appl. Genet.* 127 2333–2348. 10.1007/s00122-014-2380-1 25186168

[B13] Gonzalez F. G. Miralles D. J. Slafer G. A. (2011). Wheat floret survival as related to pre-anthesis spike growth. *J. Exp. Bot.* 62 4889–4901. 10.1093/jxb/err182 21705386

[B14] Guo J. Zhang Y. Shi W. P. Zhang B. Q. Zhang J. J. Xu Y. H. (2015). Association analysis of grain-setting rate at the apical and basal spikelets in bread wheat (*Triticum aestivum* L.). *Front. Plant Sci.* 6:1029. 10.3389/fpls.2015.01029 26635852 PMC4653486

[B15] Guo Z. F. Schnurbusch T. (2015). Variation of floret fertility in hexaploid wheat revealed by tiller removal. *J. Exp. Bot.* 19 5945–5958. 10.1093/jxb/erv303 26157170 PMC4566983

[B16] Ikeda M. Miura K. Aya K. Kitano H. Matsuoka M. (2013). Genes offering the potential for designing yield-related traits in rice. *Curr. Opin. Plant Biol.* 16 213–220. 10.1016/j.pbi.2013.02.002 23466256

[B17] Johnson E. B. Nalam V. J. Zemetra R. S. Riera-Lizarazu O. (2008). Mapping the compactum locus in wheat (*Triticum aestivum* L.) and its relationship to other spike morphology genes of the triticeae. *Euphytica* 163 193–201. 10.1007/s10681-007-9628-7

[B18] Jones B. H. Blake N. K. Heo H. Y. Martin J. M. Torrion J. A. Talbert L. E. (2021). Allelic response of yield component traits to resource availability in spring wheat. *Theor. Appl. Genet.* 134 603–620. 10.1007/s00122-020-03717-7 33146737

[B19] Klindworth D. L. Williams N. D. Joppa L. R. (1990). Chromosomal location of genes for supernumerary spikelet in tetraploid wheat. *Genome* 33 515–520. 10.1139/g90-076

[B20] Kuzay S. Lin H. Li C. Chen S. Woods D. P. Zhang J. (2022). WAPO-A1 is the causal gene of the 7AL QTL for spikelet number per spike in wheat. *PLoS Genet.* 18:e1009747. 10.1371/journal.pgen.1009747 35025863 PMC8791482

[B21] Kuzay S. Xu Y. F. Zhang J. L. Katz A. Pearce S. Su Z. Q. (2019). Identification of a candidate gene for a QTL for spikelet number per spike on wheat chromosome arm 7AL by high-resolution genetic mapping. *Theor. Appl. Genet.* 132 2689–2705. 10.1007/s00122-019-03382-5 31254024 PMC6708044

[B22] Li Y. P. Li L. Zhao M. C. Guo L. Guo X. X. Zhao D. (2021). Wheat frizzy panicle activates vernalization1-A and HOMEOBOX4-A to regulate spike development in wheat. *Plant Biotechnol. J.* 19 1141–1154. 10.1111/pbi.13535 33368973 PMC8196646

[B23] Luo W. Ma J. Zhou X. H. Sun M. Kong X. C. Wei Y. M. (2016). Identification of quantitative trait loci controlling agronomic traits indicates breeding potential of tibetan semiwild wheat (*Triticum aestivum* ssp. tibetanum). *Crop Sci.* 56 2410–2420. 10.2135/cropsci2015.11.0700

[B24] Ma J. Ding P. Y. Liu J. J. Li T. Zou Y. Y. Habib A. (2019). Identification and validation of a major and stably expressed QTL for spikelet number per spike in bread wheat. *Theor. Appl. Genet.* 132 3155–3167. 10.1007/s00122-019-03415-z 31435704

[B25] Martinek P. Bednar J. (2001). “Changes of spike morphology (multirow spike-MRS, long glumes-LG) in wheat (*Triticum aestivum*L.) and their importance for breeding,” in *Proceedings of the International Conference “Genetic Collections, Isogenic and Alloplasmic Lines”*, Novosibirsk, 192–194.

[B26] McMaster G. S. (2005). Phytomers, phyllochrons, phenology and temperate cereal development. *J. Agr. Sci.* 143 137–150. 10.1017/S0021859605005083

[B27] Millet E. (1986). Genetic control of heading date and spikelet number in common wheat (*Triticum aestivum* L.) line ‘Noa’. *Theor. Appl. Genet.* 72 105–107. 10.1007/BF00261463 24247780

[B28] Muramatsu M. (1963). Dosage effect of the spelta gene q of hexaploid wheat. *Genetics* 48:469–482. 10.1093/genetics/48.4.469 17248158 PMC1210486

[B29] Patil J. A. (1958). Inheritance study in wheat. *Curr. Sci.* 27 404–405.

[B30] Peng Z. S. Liu D. C. Yen C. Yang J. L. (1998). Genetic control of supernumerary spikelet in common wheat line LYB. *Wheat Inform. Ser.* 86 6–12.

[B31] Pennell A. L. Halloran G. M. (1984). Influence of time of sowing, photoperiod and temperature on supernumerary spikelet expression in wheat (*Triticum*). *Can. J. Bot.* 62 1687–1692. 10.1139/b84-228

[B32] Poursarebani N. Seidensticker T. Koppolu R. Trautewig C. Gawronski P. Bini F. (2015). The genetic basis of composite spike form in barley and ‘miracle-wheat’. *Genetics* 201 155–165. 10.1534/genetics.115.176628 26156223 PMC4566260

[B33] Quarrie S. A. Quarrie S. P. Radosevic R. Rancic D. Kaminska A. Barnes J. D. (2006). Dissecting A wheat QTL for yield present in a range of environments: from the QTL to candidate genes. *J. Exp. Bot.* 57 2627–2637. 10.1007/s00284-007-9035-2 16831847

[B34] Rawson H. M. (1970). Spikelet number, its control and relation to yield per ear in wheat. *Aust. J. Biol. Sci.* 23 1–15. 10.1071/bi9700001

[B35] Sakuma S. Golan G. Guo Z. F. Ogawa T. Tagiri A. Sugimoto K. (2019). Unleashing floret fertility in wheat through the mutation of a homeobox gene. *Proc. Natl. Acad. Sci. U.S.A.* 116 5182–5187. 10.1073/pnas.1815465116 30792353 PMC6421441

[B36] Sears E. R. (1954). *The Aneuploids of Common Wheat.* Columbia, IN: University of Missouri, 7–11.

[B37] Sharman B. C. (1967). Interpretation of the morphology of various naturally occurring abnormalities of the inflorescence ofwheat (*Triticum*). *Can. J. Bot.* 45 2073–2080. 10.1139/b67-224

[B38] Simons K. J. Fellers J. P. Trick H. N. Zhang Z. Tai Y. S. Gill B. S. (2006). Molecular characterization of the major wheat domestication gene Q. *Genetics* 172 547–555. 10.1534/genetics.105.044727 16172507 PMC1456182

[B39] Van-Ooijen J. W. (2006). *JoinMap^®^ 4.0: Software for the Calculation of Genetic Linkage Maps In Experimental Populations.* Wageningen: Kyazma BV.

[B40] Van-Ooijen J. W. (2009). *MapQTL^®^ 6.0: Software for the Mapping Fo Quantitative Trait Loci In Experimental Populations of Diploid Species.* Wageningen: Kyazma BV.

[B41] Voss-Fels K. P. Keeble-Gagnère G. Hickey L. T. Tibbits J. Nagornyy S. Hayden M. J. (2019). High-resolution mapping of rachis nodes per rachis, a critical determinant of grain yield components in wheat. *Theor. Appl. Genet.* 132 2707–2719. 10.1007/s00122-019-03383-4 31254025

[B42] Wang Y. Miao F. Yan L. L. (2016). Branching shoots and spikes from lateral meristems in bread wheat. *PLoS One* 11:e0151656. 10.1371/journal.pone.0151656 26986738 PMC4795765

[B43] Ward B. P. Brown-Guedira G. Kolb F. L. Van Sanford D. A. Tyagi P. Sneller C. H. (2019). Genome-wide association studies for yield-related traits in soft red winter wheat grown in Virginia. *PLoS One* 14:e0208217. 10.1371/journal.pone.0208217 30794545 PMC6386437

[B44] Würschum T. Leiser W. L. Langer S. M. Tucker M. R. Longin C. F. H. (2018). Phenotypic and genetic analysis of spike and kernel characteristics in wheat reveals long-term genetic trends of grain yield components. *Theor. Appl. Genet.* 131 2071–2084. 10.1007/s00122-018-3133-3 29959471

[B45] Xu Y. F. Wang R. F. Tong Y. P. Zhao H. T. Xie Q. G. Liu D. C. (2014). Mapping QTLs for yield and nitrogen-related traits in wheat: influence of nitrogen and phosphorus fertilization on QTL expression. *Theor. Appl. Genet.* 127 59–72. 10.1007/s00122-013-2201-y 24072207

[B46] Yang W. Y. Lu B. R. Hu X. R. Yu Y. Zhang Y. (2005). Inheritance of the triple-spikelet character in a Tibetan landrace of common. *Genet. Resour. Crop Evol.* 52 847–851. 10.1007/s10722-003-6089-2

[B47] Zhai H. J. Feng Z. Y. Li J. Liu X. Y. Xiao S. H. Ni Z. F. (2016). QTL analysis of spike morphological traits and plant height in winter wheat (*Triticum aestivum* L.) using a high-density SNP and SSR-based linkage map. *Front. Plant Sci.* 7:1617. 10.3389/fpls.2016.01617 27872629 PMC5097907

[B48] Zhang J. L. Gizaw S. A. Bossolini E. Hegarty J. Howell T. Carter A. H. (2018). Identification and validation of QTL for grain yield and plant water status under contrasting water treatments in fall-sown spring wheats. *Theor. Appl. Genet.* 131 1741–1759. 10.1007/s00122-018-3111-9 29767279 PMC6061171

[B49] Zhang R. Q. Hou F. Chen J. Chen S. L. Xing L. P. Feng Y. G. (2017). Agronomic characterization and genetic analysis of the supernumerary spikelet in tetraploid wheat (*Triticum turgidum* L.). *J. Integ. Agric.* 16 1304–1311. 10.1016/S2095-3119(16)61469-7

[B50] Zhang R. Q. Wang X. E. Chen P. D. (2013). Inheritance and mapping of gene controlling four-rowed spike in tetraploid wheat (*Triticum turgidum* L.). *Acta. Agron. Sin.* 39 29–33. 10.3724/SP.J.1006.2013.00029

